# Effect of pH-Dependent Homo/Heteronuclear CAHB on Adsorption and Desorption Behaviors of Ionizable Organic Compounds on Carbonaceous Materials

**DOI:** 10.3390/ijerph191912118

**Published:** 2022-09-25

**Authors:** Xiaoyun Li, Jinlong Zhang, Yaofeng Jin, Yifan Liu, Nana Li, Yue Wang, Cong Du, Zhijing Xue, Nan Zhang, Qin Chen

**Affiliations:** 1 Department of Environmental Science, School of Geography and Tourism, Shaanxi Normal University, Xi’an 710119, China; 2 International Joint Research Centre of Shaanxi Province for Pollutants Exposure and Eco-Environmental Health, Xi’an 710119, China; 3 Environmental Protection Department of Mahe Town, Yuyang District, Yulin 719000, China; 4 Northwest Land and Resource Research Center, Shaanxi Normal University, Xi’an 710119, China

**Keywords:** ionizable organic chemicals, hydrogen bond, desorption hysteresis, carbonaceous materials, density functional theory

## Abstract

Herein, the adsorption/desorption behaviors of benzoic acid (BA) and phthalic acid (PA) on three functionalized carbon nanotubes (CNTs) at various pH were investigated, and the charge-assisted H-bond (CAHB) was verified by DFT and FTIR analyses to play a key role. The results indicated that the adsorption order of BA and PA on CNTs was different from *K*_ow_ of that at pH 2.0, 4.0, and 7.0 caused by the CAHB interaction. The strength of homonuclear CAHB (≥78.96 kJ·mol^−1^) formed by BA/PA on oxidized CNTs is stronger than that of heteronuclear CAHB formed between BA/PA and amino-functionalized CNTs (≤51.66 kJ·mol^−1^). Compared with the heteronuclear CAHB (Hysteresis index, HI ≥ 1.47), the stronger homonuclear CAHB leads to clearly desorption hysteresis (HI ≥ 3.51). Additionally, the contribution of homonuclear CAHB (≥52.70%) was also greater than that of heteronuclear CAHB (≤45.79%) at pH 7.0. These conclusions were further confirmed by FTIR and DFT calculation, and the crucial evidence of CAHB formation in FTIR was found. The highlight of this work is the identification of the importance and difference of pH-dependent homonuclear/heteronuclear CAHB on the adsorption and desorption behaviors of ionizable organic compounds on carbonaceous materials, which can provide a deeper understanding for the removal of ionizable organic compounds by designed carbonaceous materials.

## 1. Introduction

Ionizable organic compounds (IOCs), such as antibiotics, pesticides, endocrine disrupting compounds, perfluoroalkyl substances, and many personal care products, are produced on a large scale and widely used in industry, agriculture and everyday life [1,2]. Therefore, release of IOCs into the environment is inevitable, and many of them are widely detected in industrial effluents, surface and ground waters [3,4,5]. Due to their relatively high hydrophilicity, it is easy for IOCs to migrate and transform in the natural environment [1,6,7], thus causing potential toxic effects on aquatic organisms and human health through the food chain [8,9]. Therefore, it is very necessary and urgent to better understand the fate of IOCs, and further to effectively remove IOCs from the aquatic environment [1,10,11]. 

Adsorption as a highly efficient and environmentally friendly method of pollutant removal has been widely used in the removal of IOCs in water [12,13]. It is very common for multiple pH-dependent IOC species to coexist in the same environmental system, and pH would play a vital key in regulating the proportion of their existing forms [14,15]. Thus, the adsorption behavior of IOCs could vary significantly across a broad scale depending on a wide pH range of natural water bodies from 2.2~9.8 [8,16,17]. Numerous studies have shown that the adsorption mechanisms of IOCs on carbonaceous materials mainly include hydrophobic, π–π electron donor–acceptor (π–π EDA), electrostatic and hydrogen bond (H-bond) interactions, and different mechanisms may act simultaneously [18,19,20]. As for H-bond, previous studies mainly focused on ordinary H-bond [21,22,23], namely weak electrostatic interactions caused by permanent and instantaneous dipoles of molecules. Recently, Ni et al. [24] first proposed a negative charge assisted hydrogen bonding (CAHB) between aromatic carboxylate ions and biochar, and then the CAHB adsorption mechanism has been gradually verified and widely assumed in subsequent studies between IOCs and functionalized adsorbents [25,26,27,28,29]. CAHB is much stronger and more stable than ordinary H-bond, and its strength increases with decreasing |Δp*K*_a_| (<5.0) between the hydrogen donor and acceptor [30,31]. This CAHB adsorption theory provides a new perspective for further understanding the environmental behavior of IOCs. However, to date, most of the studies on CAHB adsorption mechanism have only focused on the observation of apparent phenomena (such as the rising of the solution pH) [24], thermodynamic theoretical analysis [26,32], and density functional theory (DFT) calculation [29]. Additionally, the calculation of DFT only considered O—H···O (homonuclear H-bond), while there is a lack of discussion on N—H···O/O—H···N (heteronuclear H-bond). In addition, the spectral evidence for homonuclear and heteronuclear CAHB formation at the molecular scale still needs further probing. 

The existing species of IOCs are mainly dependent on the solution pH. When pH < the lowest p*K*_a_ – 2 or pH > the highest p*K*_a_ + 2, only one IOCs species is dominant in the solution. Thus, the adsorption behavior of the IOCs is controlled by the dominated IOC species. However, when at intermediate pH, two or more than two types of IOC species co-exist in the same system, and the whole adsorption of IOCs becomes a combined performance of different species. Up to the present, it is still a challenge to accurately calculate the adsorption contributions of different IOC species at a pH nearby the p*K*_a_ of IOCs [14]. In addition, except for pH and Δp*K*_a_ values between the donor and acceptor, homonuclear (O—H···O) or heteronuclear H-bond (N—H···O/O—H···N) may also affect the adsorption affinity of IOCs on adsorbents, thereby influencing their environmental behavior. In order to comprehend the adsorption mechanism and environmental behavior of IOCs even better, three different types of functionalized multiwalled carbon nanotubes (CNTs) were selected as the representative material in the present study to explore the effects of pH, Δp*K*_a,_ and homonuclear/heteronuclear H-bond properties on CAHB formation between IOCs and carbonaceous materials. 

Benzoic acid (BA) and phthalic acid (PA), because of their similar molecular structure and different p*K*_a_ values, are chosen as representatives for IOCs. BA is commonly used as food additives or flavoring agents in pharmaceutical and cosmetic products [33]. PA is also an important organic industrial product, and its derivatives are widely used in plasticizers, synthesis dyes, polyester resins, polyester fibers, medicines, and personal care products [34]. BA and PA have both been frequently detected in groundwater, surface water, and surface soil [35,36,37,38]. Previous studies pointed out that both BA and PA would have adverse effects on human health and the water environment [33,39,40,41]. 

The aims of this study are (1) to illustrate the dominant adsorption mechanism of BA and PA on different functionalized CNTs under various pHs; (2) to obtain spectral evidence for homonuclear and heteronuclear CAHBs formation between BA/PA and CNTs; (3) to explore the effect of homonuclear/heteronuclear CAHB and solution pH on adsorption behaviors of IOCs by CNTs; (4) to further clarify the adsorption mechanism and configuration of different CAHBs by DFT calculation. This study will further deepen our understanding of the role and influence of CAHB in the environmental behavior of IOCs and provide new directions for the management of IOCs in wastewater.

## 2. Materials and Methods

### 2.1. Adsorbents, Chemicals, and Characterization

Three types of zigzag multi-walled CNTs, including oxygen-rich (O-CNTs), amino-rich (N-CNTs), and graphitized carbon nanotubes (G-CNTs), were obtained from Chengdu Organic Chemicals Co. Ltd., Chinese Academy of Sciences. All used CNTs were characterized for their surface morphologies with TEM (JEM-2100, Japan) and SEM (FEI Quanta 200, USA). The total elemental composition and the surface elemental composition were analyzed by an elemental analyzer (Vario ELIII, Germany) and XPS (AXIS-ULTRA, Japan), respectively. A full-automatic surface area and pore distribution analyzer (ASAP2460, Micromeritics, USA) was used for the surface area determination at 77 K, and surface functional groups were examined by FTIR (Tensor 27, Bruker, Germany) at 298.15 K with the range of 4000–400 cm^−1^. The ξ-potential was determined with a Nano Zeta Sizer (Bi-90 plus, Brookhaven, USA), and the buffering capability was also determined independently by titration with 0.2 g CNTs (pH from 2.0 to 12.0). All the characterization processes and results were displayed in Text S1 and Table S1, respectively. BA and PA (purity ≥ 99%) were bought from Sigma-Aldrich and utilized without any other process. Selected properties of adsorbates were presented in Table S2. Other chemicals and reagents used in the experiment, such as NaN3, HCl, NaOH, NaCl, and acetonitrile, were all guaranteed reagents. All experiments utilized Milli-Q water. 

### 2.2. Bath Adsorption and Desorption Experiments at Different pHs

The batch equilibrium adsorption assays of BA and PA on three types of CNTs were carried out in 8 mL glass vials. 200 mg·L^−1^ NaN3 (biocides) and 0.02 mol·L^−1^ NaCl were added to ultra-pure water to prepare the background solution, and then the solutions of BA or PA at different concentrations (0~150 mg·L^−1^) were generated in the background solution, and the pH was adjusted to 2.0, 4.0, or 7.0 using HCl or NaOH. An amount of 50 mg CNTs was first pre-wetted in 1 mL of background solution with the same pH for 48 h in a glass vial as the desired concentration of BA/PA solution. Following that, 7 mL of the required adsorbate solution was added, and the mixed vials were shaken for another 48 h (no significant difference after 48 h, Figure S1) in the dark (25 ± 1 °C) at 180 rpm. After centrifugation (3000 rpm for 30 min), the supernatant firstly passed through the micropore membrane filter (0.45 μm) and then the concentration of adsorbates was determined via HPLC (Thermo U3000, USA) with a C18 column at 226 nm and 231 nm for BA and PA, respectively. All adsorption tests were conducted at least twice. 

Desorption tests were only performed at pH 2.0 and 7.0. After reaching the adsorption equilibrium (48 h), 4 mL of the supernatant was removed and then 4 mL of background solution with the same pH was added. The vials were sealed and continued to shake for an additional 48 h (Figure S1), and then sampled in accordance with the instructions provided in adsorption tests. The supernatant concentration of BA/PA was measured by HPLC. The desorption number of IOCs was computed according to the concentration difference before and after desorption. All desorption assays were carried out with two parallels. 

Adsorption kinetics of BA and PA on O-CNTs at pH 7.0 was investigated by using batch equilibration technique and all the experiments were performed with at least two replications. A previous study indicated that the effect of equilibrium time on the initial environmental factors was negligible [42]. Therefore, the initial concentration of BA or PA was 50 mg·L^−1^ to determine adsorption kinetics. The contact time was set to 1 h, 5 h, 10 h, 24 h, 48 h, and 72 h, respectively. Each sample was centrifuged, filtered, and determined by HPLC immediately after taking it out. 

### 2.3. Data Analysis

The adsorption kinetic data were fitted employing the pseudo-first-order, pseudo-second-order, and intra-particle diffusion models, and the isothermal adsorption data were fitted by the Freundlich and Langmuir models. The optimal fitting model was considered using lower sum of squares residual (SSR) values and higher adjusted square of correlation coefficient (Adj r^2^) values. The adsorption affinity of IOCs on CNTs could be described through the distribution coefficient (K_d_). All of the above-mentioned formulas and analysis procedures were fully described in Text S2. 

The desorption extent was reflected by release ratio (R_r_) [43] and hysteresis index (HI) [44]:

(1) $$R_{r}\;(\%\;\mathrm{of}\;\mathrm{adsorbed})=\;\frac{Q_{e}-Q_{\mathrm{desorption}3}}{Q_{e}}\times 100\%$$

(2) $$\mathrm{Hysteresis}\;\mathrm{index}\;(\mathrm{HI})=\frac{Q_{e}^{d}-Q_{e}^{s}}{Q_{e}^{s}}{│}_{T,C_{e}}$$

where Q_e_ (mg·g^−1^) and Q_desorption3_ (mg·g^−1^) are the equilibrium adsorption concentration and the equilibrium adsorption concentration after the third desorption step. $Q_{e}^{d}$ (mg·g^−1^) is solid-phase solute concentration at desorption equilibrium, and $Q_{e}^{s}$ (mg·g^−1^) is the solid-phase solute concentration derived from the liquid-phase solute concentration C_e_ (mg·L^−1^) depending on the assumption that adsorption is totally reversible. The closer HI is to 0, the lower degree of desorption hysteresis is. The subscripts T and C_e_ denote the temperature and solute equilibrium concentration respectively. 

### 2.4. DFT Computational Details

DFT calculation was carried out to better discern the homonuclear/heteronuclear CAHB interaction between adsorbates and CNTs. The B3LYP functional and the 6-311 + G** basis set were used in the Gaussian 09W software package for energy computation and geometric optimization, and the DFT-D3(BJ) dispersion correction was performed [29]. The polarizable continuum model (PCM) implicit solvent model (water) was employed to account for solvation effects. The model of G-CNTs that is typically employed is the armchair CNTs (3, 3) with 48 C atoms [45,46]. Moreover, the number of functional groups on the tips is usually more than that on the sidewalls of CNTs because of the highest defect sites on the tips [47,48]. Hence, O-CNTs and N-CNTs were simulated by attaching functional groups of –OH/–COOH and –NH2 to the tips of the G-CNTs. The optimized structures of neutral O-CNTs, N-CNTs, and IOCs are shown in Figure S2. The binding energy (E_ads_) of homonuclear/heteronuclear CAHB formed between IOCs and O-CNTs/N-CNTs was calculated via Equation (3) [46,49]:

E_ads_ = E _(CNTs+IOCs)_ − E_CNTs_ − E_IOCs_

where E_CNTs+IOCs_ represents the total energy of IOCs on CNTs, E_CNTs_ is the total energy of CNTs, E_IOCs_ is the total energy of IOCs. The obtained E_ads_ was also corrected by equilibrium correction method considering the basis set superposition error (BSSE). 

## 3. Results and Discussion

### 3.1. Characterization of CNTs

**Figure 1:**
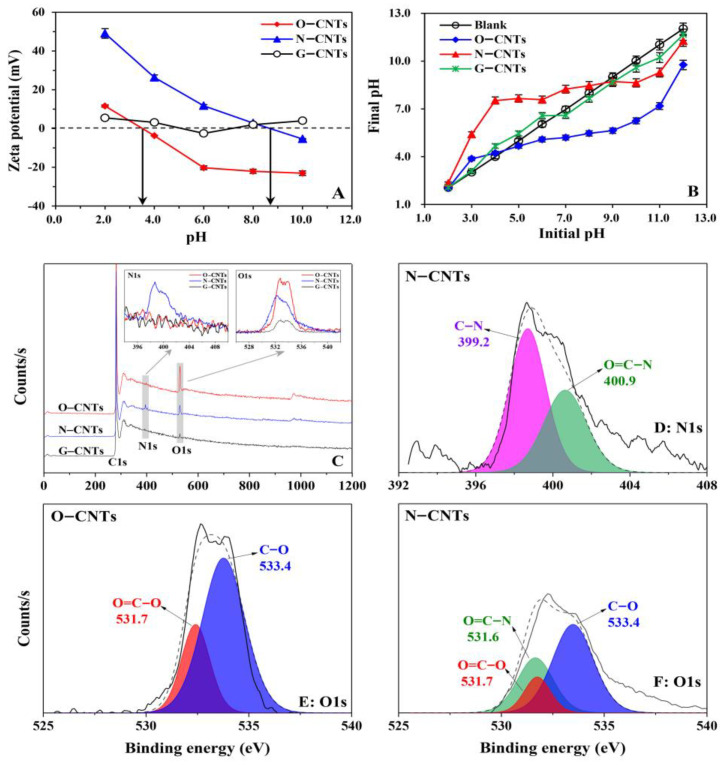
(**A**): Zeta potential of the adsorbents in aqueous solution. The data points in the graph were averaged using three parallel sets. (**B**): Titration curves of the three adsorbents. Blank is background solution only with the same amount of HCl/NaOH as to the samples. (**C**): XPS spectra and high-resolution spectra (O1s and N1s) for O-CNTs, N-CNTs, G-CNTs (survey scans the spectral region from 0 to 1200 eV). Peaks fitting for N1s (**D**: N-CNTs) and O1s (**E**: O-CNTs, **F**: N-CNTs).

The TEM (A, B, and C) and SEM (D, E, and F) images of three CNTs are presented in Figure S3. It can be found that the surface roughness of O-CNTs and N-CNTs was more obvious than that of G-CNTs, and CNTs tubes were obviously bent or broken due to structural defects after oxidized or ammoniated modification, thereby leading to their specific surface area increase in the order (Table S1) of G-CNTs (66.87 m^2^·g^−1^) < O-CNTs (81.50 m^2^·g^−1^) < N-CNTs (172.61 m^2^·g^−1^). In addition, the longer chain of amine provided larger pore volume is also an important reason for the significantly larger specific surface area of N-CNTs, especially the pore volume within the range of 9–76 nm (Figure S4). The presence of the different functional groups on the CNTs surface causes a change in the pH_PZC_ of the adsorbents [42]. The pH_pzc_ of O-CNTs and N-CNTs are 3.07 and 8.76, respectively (Figure 1A). The lack of functional groups on the G-CNTs surface is likely responsible for its unstable PZC. The titration results of the buffering capability of different CNTs are shown in Figure 1B. The pH_PZC_ of the tested CNTs was determined by taking the intersection of the lines pH (final) = pH (initial), which is similar to those of the zeta potential discussed above. Compared to the G-CNTs (6.60), the decline in the pH_PZC_ of the O-CNTs (3.65~4.22) is mainly because of the introduction of the acidic O-groups on the surface. However, the introduction of amino groups on the CNTs surface results in an increase in pH_PZC_ of N-CNTs (8.46~8.67). 

The peaks fitting of O 1s spectra (Figure 1E,F and Figure S5) display that peaks at 531.6, 531.7, and 533.4 eV correspond to O=C-N, O=C-O, and C-O, respectively [18,43]. The two peaks at 399.2 eV and 400.9 eV are ascribed to the groups of C-N and O=C-N [44], respectively, which showed up in the peaks fitting of N 1s spectra (Figure 1D). To further gain more information about the surface functional groups on CNTs, FTIR spectra of the three CNTs are recorded and depicted in Figure S6. The strong peaks at 3465 and 1640 cm^−1^ in O-CNTs and N-CNTs can be assigned to the stretching vibration of the O–H and C=O groups. The weak peaks at 3465 and 1640 cm^−1^ also appear in G-CNTs, which is consistent with the above XPS result. In addition, the FTIR spectra of N-CNTs show two bands at 3433 and 1570 cm^−1^, corresponding to the in-plane bending mode of the primary amine NH_2_ and the bending mode of the secondary amine N–H, respectively, further demonstrating the existence of the amide functional groups on N-CNTs surface. The band at 3160 cm^−1^ is considered the red-shift peak of NH_2_, which is attributed to the overlapping bands of the NH_2_ and O–H [45]. A broad band between 616~810 cm^−1^ is caused by the out-of-plane NH_2_ bending mode [46]. Another important new adsorption band is the stretching of the C–N bond present in N-CNTs at 1142 cm^−1^, which further indicates the successful formation of the saturated primary amine on the CNTs surface. 

### 3.2. Adsorption Kinetics of BA and PA on CNTs

**Figure 2:**
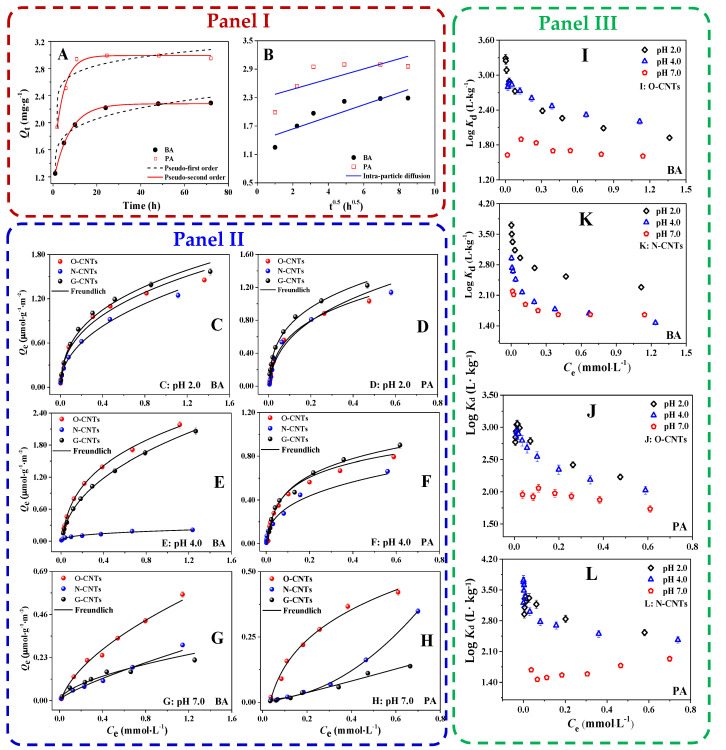
Panel I: The fitting results for (**A**) pseudo-first order, pseudo-second order and (**B**) intra-particle diffusion kinetic models of BA and PA on O-CNTs. Cinitial = 50 mg·L^−1^, pH = 7.0, T = 25 °C, m/V = 0.625 g·L^−1^, contact time = 72 h. Panel II: Adsorption isotherms of BA (**C**,**E**,**G**) and PA (**D**,**F**,**H**) on CNTs at different pHs after surface area normalization. Panel III: Log*K*_d_ values of BA (**I**,**K**) and PA (**J**,**L**) on O-CNTs and N-CNTs with increasing equilibrium concentration at different pH conditions. Error bars may be covered by symbol.

Adsorption kinetics and adsorption equilibrium time of BA and PA on O-CNTs at pH 7.0 were evaluated (Figure 2 Panel I). The adsorption capacities of both BA and PA go up with increasing contact time, and 48 h is sufficient for adsorption equilibrium of BA and PA on O-CNTs to be achieved. There are three distinct stages to the adsorption process. In the first stage, the adsorption amount (Q_t_) increased rapidly within the initial 10 h owing to the adequate adsorption sites on the surface of O-CNTs for removal of BA and PA. Then, in the second stage within the 10~24 h, it proceeded at an obviously slower rate and finally reached saturation at 48 h. 

The adsorption kinetics results fitting by the pseudo-first-order and pseudo-second-order models are shown in Figure 2A and Table S3. According to the bigger correlation coefficient Adj r^2^ (0.9998 for BA and 0.9997 for PA) and the smaller SSR (0.758 for BA and 0.953 for PA), and the closer values between the experimental adsorption capacity Q_e,exp_ (BA: 2.29 mg·g^−1^, PA: 2.96 mg·g^−1^) and the maximum capacity Q_e,cal_ calculated by the pseudo-second-order model (BA: 2.34 mg·g^−1^, PA: 3.00 mg·g^−1^), the pseudo-second-order model is found to be more accurate for comprehending the adsorption kinetic data of both BA and PA on O-CNTs. These results imply that the adsorption rate was mainly controlled by chemical processes performed on the O-containing functional group sites of the O-CNT surface [47]. In other words, the chemical adsorbent-adsorbate interaction is predominantly responsible for the adsorption of BA and PA on O-CNTs at pH 7.0. Notably, the adsorption rate (k_2_) of PA (70.95 g·mg^−1^·h^−1^) on O-CNTs was more than 8 times higher than that of BA (8.71 g·mg^−1^·h^−1^) on O-CNTs, which may be due to the fact that Δp*K*_a_ between PA and O-CNTs is less than 3.0, thus two COOH (pK_a1_ = 2.98, p*K*_a2_ = 5.28, Table S2) on PA could both generate a strong CAHB interaction with O- groups on O-CNTs surface (PZC≈3.07, Table S1), leading to a remarkable augment in the adsorption rate. 

To identify the main rate-controlling step, the intra-particle diffusion model was further employed to analyze the kinetic data of BA and PA on O-CNTs. According to Figure 2B, the linear plots of Q_t_ vs. t^0.5^ not only did not cross the origin and appeared to be multi-linear, but also had very poor linear regression coefficients (Adj r^2^: 0.7376 for BA and 0.4622 for PA, Table S3), which indicates that this model was not applicable, and there must be an additional rate-controlling step in addition to intra-particle diffusion [47,48]. It may be concluded that during the adsorption process, both intra-particle diffusion and external surface adsorption were active simultaneously. In addition, the values of C (the intercept of the Q_t_ vs. t^0.5^ plots) can provide some useful information. The external surface adsorption becomes more prominent as the rate-limiting step when the value of C is higher [47]. Regarding the values of C (1.38 mg·g^−1^ for BA and 2.26 mg·g^−1^ for PA) in this study, the external surface diffusion has a greater impact on the adsorption rate of PA on O-CNTs than that of BA on O-CNTs, which is consistent with the above conclusion that both COOH of PA could involve in a strong CAHB surface interaction with O-groups of O-CNTs.

### 3.3. Adsorption Isotherms of BA and PA on CNTs-Effect of pH

In this study, isothermal adsorption experiments were performed under pH 2.0, 4.0, and 7.0. In order to more accurately evaluate the impacts of various surface functional groups (e.g., –OH, –COOH, –NH_2_) on the CNTs adsorbing BA and PA, all equilibrium adsorbed concentrations (Q_e_) were normalized to a specific surface area. The equilibrium adsorption data were analyzed by the Freundlich and Langmuir models (Figure 2 Panel II and Table S4), respectively. In terms of the greater Adj r^2^ (≥ 0.9488) and lower SSR (≤ 0.0177) values of the Freundlich model than that of the Langmuir model (Adj r^2^ ≥ 0.8696, SSR ≤ 0.0581), and some abnormal Q_0_ values of the Langmuir model, the Freundlich model is selected to fit the data of BA and PA on different CNTs at various pHs. 

As seen from Figure 2 Panel II, adsorption of BA and PA on various CNTs after normalization to a specific surface area at different pH conditions revealed different trends. At pH 2.0, both BA (>99%, p*K*_a_ = 4.17, Figure S7) and PA (>92%, p*K*_a1_ = 2.98, p*K*_a2_ = 5.28) predominantly existed as molecules. Their relatively large hydrophobicity (Log*K*_ow_ = 1.63 for BA and Log*K*_ow_ = 1.24 for PA, Table S2) at pH 2.0 resulted in the highest adsorption capacity of BA and PA on G-CNTs. For BA, the higher adsorption amount on O-CNTs per unit surface area than that on N-CNTs may be due to the repulsion (between N-CNTs and BA···H_3_O^+^) caused by the greater electro-positivity of N-CNTs (PZC ≈ 8.76, Figure 1). For PA, about 8% of them existed as PA^−1^, which could interact with O-CNTs and N-CNTs by electrostatic attraction and H-bond except for the hydrophobic effect. When the solution pH increased to 4.0, about 48% of BA was converted to BA^−1^. Due to the decrease in hydrophobicity caused by this transformation, the adsorption amount of BA on O-CNTs should also be reduced. In fact, the adsorption amount of BA on O-CNTs per unit surface area not only did not decrease, but also obviously increased instead. According to our previous research, this is mainly because the strong homonuclear CAHBs [CNTs-Oˉ···H^+^···ˉOOC-BA or CNTs-COOˉ···H^+^···ˉOOC-BA] were formed between BA-1 and O-CNTs due to their small |∆p*K*_a_| = |PZC_O-CNTs_ – p*K*_a_,_BA_|≈ 0.52 [18]. However, the adsorption of BA on N-CNTs was markedly reduced. Although in theory, the weak heteronuclear CAHBs [CNTs-NH_2_···H^+^···ˉOOC-BA] can also be formed between N-CNTs and BA, the contribution of this interaction is very limited due to the large |∆p*K*_a_| = |PZC_N-CNTs_ – p*K*_a_,_BA_|≈ 4.59 between BA and N-CNTs. While for PA, about 88% of PA existed in PA^−1^ forms at pH 4.0. An interesting observation is that the adsorption of PA on N-CNTs per unit surface area did not decrease as significantly as that of BA, which should be caused by the electrostatic attraction and the heteronuclear CAHBs formed between PA and N-CNTs due to their relatively smaller |∆p*K*_a_| = |PZC_N-CNTs_ – p*K*_a_,_PA_|≈ 3.48. At pH 7.0, > 99% of BA and > 96% of PA existed in the form of BA^−1^ and PA^2−^, respectively, and the surface of O-CNTs was negatively charged as well, while N-CNTs were positively charged. In theory, the adsorption of both BA and PA on O-CNTs should be the lowest because of electrostatic repulsion between them, while their adsorption on N-CNTs should be the highest on account of electrostatic attraction. However, the fact is that the adsorption amount of both BA and PA on O-CNTs was the highest (Figure 2G,H), which indicates that the smaller ∆p*K*_a_ between BA/PA and O-CNTs makes the strength of the homonuclear CAHB much stronger than that of the heteronuclear CAHB between BA/PA and N-CNTs.

To further explore the adsorption mechanism of BA and PA on CNTs at different pHs, the change in distribution coefficient (*K*_d_) was employed to understand the key role of CAHB in the adsorption process. A previous study has shown that the *K*_d_ could accurately understand the adsorption capacity of pollutants on carbon nanomaterials [11]. As shown in Figure 2 Panel III, the trends of Log*K*_d_ for BA and PA on O-CNTs and N-CNTs are different at various pHs. For O-CNTs, with the pH increase from 2.0 to 4.0, the Log*K*_ow_ of BA and PA clearly decreased (Table S2), but the Log*K*_d_ values of BA and PA on O-CNTs almost did not change. Obviously, the unreduced Log*K*_d_ at pH 4.0 compared to pH 2.0 is attributable to the formation of strong homonuclear CAHBs between BA^−1^/PA-1 (Figure S7) and O-containing functional groups of O-CNTs. This strong CAHB interaction could counteract the effect of hydrophobicity reduction. However, for N-CNTs, the Log*K*_d_ values of BA on N-CNTs decreased sharply with the increase in pH from 2.0 to 4.0. On the one hand, the contribution of CAHB between BA and N-CNTs was limited due to the larger ∆p*K*_a_ ≈ 4.59. On the other hand, due to the formation of heteronuclear CAHB between BA and N-CNTs, the weak adsorption energy of heteronuclear CAHB could not offset the effect of hydrophobicity reduction (see Section 3.5 for detailed discussion). Interestingly, the Log*K*_d_ values of PA on N-CNTs did not decrease significantly with the increase in pH from 2.0 to 4.0. This is due to the smaller ∆p*K*_a_ ≈ 3.48 between PA and N-CNTs, which could form a relatively strong heteronuclear CAHB. Notably, at pH 7.0, the Log*K*_d_ values of BA and PA on both O-CNTs and N-CNTs are the lowest due to the extremely low hydrophobicity of BA and PA (Log*K*_ow_ = −1.20 for BA and Log*K*_ow_ = −3.65 for PA, Table S2). However, at pH 7.0, more than 99% BA^−1^ and more than 96% PA^−2^ could form homonuclear/heteronuclear CAHB with the functional groups on the surface of O-CNTs and N-CNTs. This strong CAHB interaction may lead to irreversible adsorption, and the specific analysis would be discussed in the next section. 

### 3.4. Desorption of BA and PA on CNTs at Different PHs

**Figure 3:**
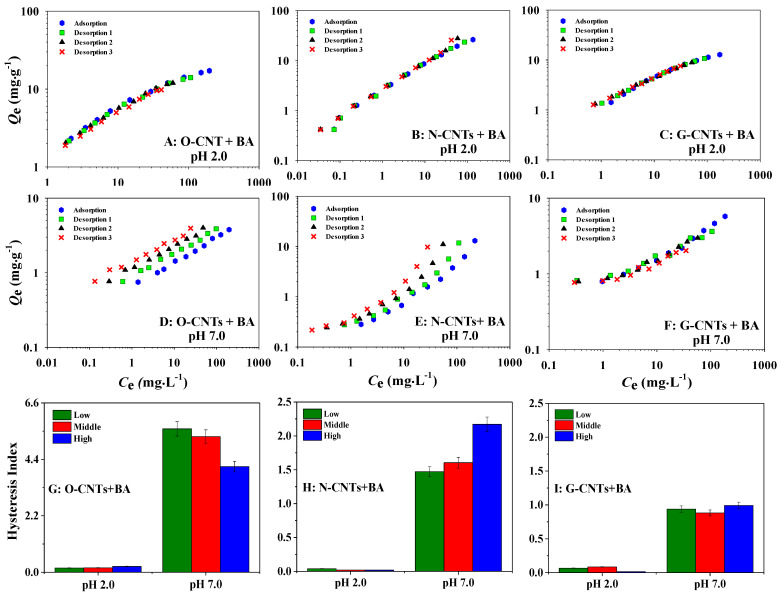
Adsorption and desorption of BA on O-CNTs, N-CNTs and G-CNTs at pH 2.0 (**A**–**C**) and pH 7.0 (**D**–**F**), respectively. And the comparison of the hysteresis index of BA (Low, Middle and High represent 5 mg·L^−1^, 80 mg·L^−1^ and 160 mg·L^−1^ of BA) on three types of CNTs (**G**–**I**) at pH 2.0 and pH 7.0. At Ph 2.0, there is no obvious desorption hysteresis of BA on three CNTs, but the obvious desorption hysteresis of BA on O-CNTs and N-CNTs occurred at pH 7.0. The hysteresis index of BA on O-CNTs (≥4.12) and N-CNTs (≥1.47) is higher than that of BA on G-CNTs (≤0.99) at pH 7.0.

At pH 2.0, 99% BA and 92% PA are molecular states, while at pH 7.0, 99% BA and 96% PA exist in anion form (Figure S7). The main adsorption mechanism of BA/PA on CNTs was significantly different due to their different existence forms at different pHs. In order to investigate the difference in main adsorption mechanisms between BA/PA in molecular and anionic forms on CNTs, three adsorption–desorption cycles were carried out at pH 2.0 and pH 7.0 to further explore the contribution of CAHB to the adsorption of BA (Figure 3) and PA (Figure S8) by CNTs. The degree of irreversible adsorption was expressed by the hysteresis index (HI, Table S5). The smaller the HI values, the lower degree of desorption hysteresis [49]. At pH 2.0, because BA and PA mainly existed in molecular states, ordinary H-bond, π–π, and hydrophobic interactions should be the main adsorption mechanisms, while CAHB had little contribution to the adsorption process. Thus, there is no obvious desorption hysteresis on all three CNTs at various concentrations (HI ≤ 0.23, Figure 3 and Figure S8, Table S5). However, at pH 7.0, except for G-CNTs, BA^−1^ and PA^−2^ showed apparent desorption hysteresis of varying degrees on both O-CNTs (HI ≥ 3.51, Figure 3G and Figure S8G, Table S5) and N-CNTs (HI ≥ 1.47, Figure 3H and Figure S8H, Table S5). It may be because ionic state of BA and PA at pH 7.0 could form homonuclear/heteronuclear CAHB on O-CNTs and N-CNTs, resulting in irreversible adsorption. However, for G-CNTs, the insignificant hysteresis (HI ≤ 0.99, Figure 3I and Figure S8I, Table S5) may be attributed to the limited contribution of CAHB due to a trace group on its surface. These results are consistent with the discussion in the above section and well confirm the important role of CAHB in the adsorption of IOCs on functionalized CNTs. 

Moreover, the contribution of CAHB in the process of adsorption and desorption was quantitatively calculated by release ratio (R_r_) (Table S5). At pH 2.0, the main adsorption mechanisms of BA/PA on all three CNTs were ordinary H-bond, π–π, and hydrophobic interactions. Therefore, the fluctuation range of Rr values was not large (35.53%~56.69%). However, at pH 7.0, the Rr values of BA/PA on all three CNTs fluctuated greatly (1.75%~59.27%), which could be explained by the CAHB adsorption theory. On the one hand, the anion state of BA/PA could form homonuclear/heteronuclear CAHB with O-CNTs and N-CNTs, resulting in lower Rr values (≤17.51%). On the other hand, the main adsorption mechanisms of BA/PA on G-CNTs were π–π and hydrophobic interactions because of the lack of functional groups on G-CNTs, resulting in the increase in Rr values to 59.27%. Meanwhile, it was found that the contribution of homonuclear CAHB (BA: 54.17% and PA: 52.70%) was greater than that of heteronuclear CAHB (BA: 42.55% and PA: 45.79%) at pH 7.0 (Table S5). This may be because the ∆p*K*_a_ absolute values between BA (0.52)/PA (0.67) and O-CNTs were smaller than that between BA (4.59)/PA (3.48) and N-CNTs (Tables S1 and S2), leading to the greater intensity of homonuclear CAHB than that of heteronuclear CAHB. Hence, the different contributions were determined by the strength of CAHB, which would be further discussed in the next section. These results show that different species of IOCs lead to different mechanisms under the regulation of pH, which is very important to control the pollution tendency of IOCs.

### 3.5. XPS, FTIR, and DFT Analysis of Homonuclear/Heteronuclear CAHB

**Figure 4:**
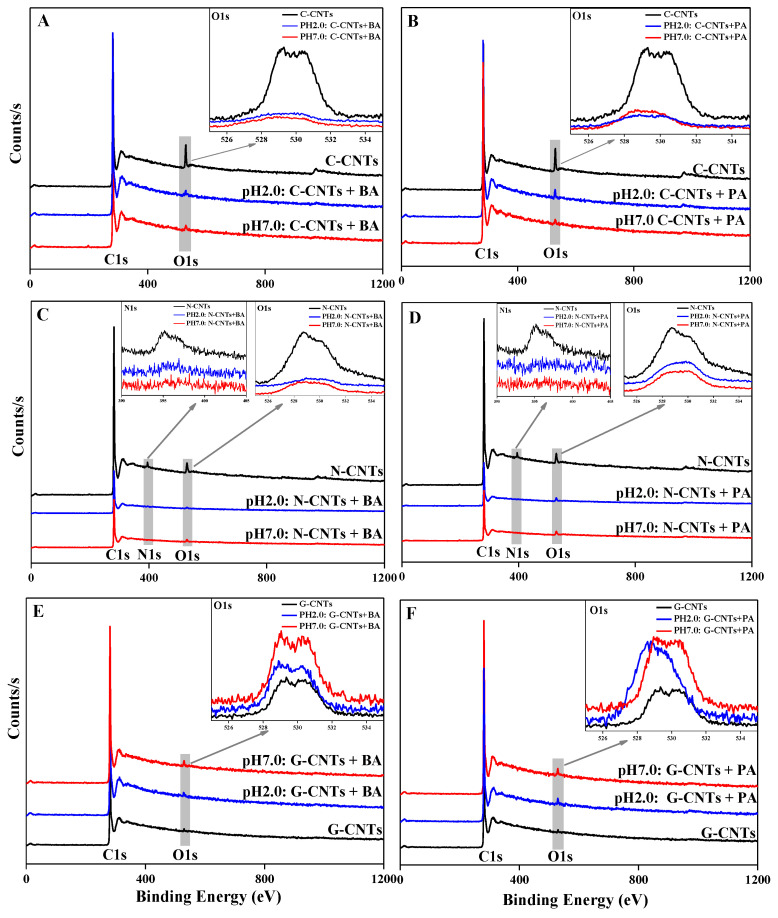
The XPS spectra of O-CNTs (**A**,**B**), N-CNTs (**C**,**D**), and G-CNTs (**E**,**F**) with loaded and unloaded IOCs.

In order to explore the spectral evidence of homonuclear/heteronuclear CAHB in molecular structure and compare the strength of homonuclear/heteronuclear CAHB, XPS, FTIR, and DFT analyses were further performed to verify the above adsorption mechanism. The XPS spectra of three CNTs with loaded and unloaded IOCs at pH 2.0 and 7.0 were compared and the results showed in Figure 4. It can be clearly seen from Figure 4A–D that, after adsorption of BA or PA at both pH 2.0 and 7.0, the contents of O and N functional groups on the O-CNTs and N-CNTs surface were both significant decreases, which can be attributed to these surface O and N functional groups involved into the hydrogen bond interactions between BA/PA and O-CNTs/N-CNTs. Conversely, the content of O groups on the G-CNTs surface was increased. This is mainly because the lack of functional groups on the surface of G-CNTs led to BA/PA adsorption on the surface of G-CNTs mainly through π–π and hydrophobic interactions. Therefore, the O groups of BA/PA were exposed on the surface, resulting in the increase in O content of G-CNTs with loaded BA/PA.

**Figure 5:**
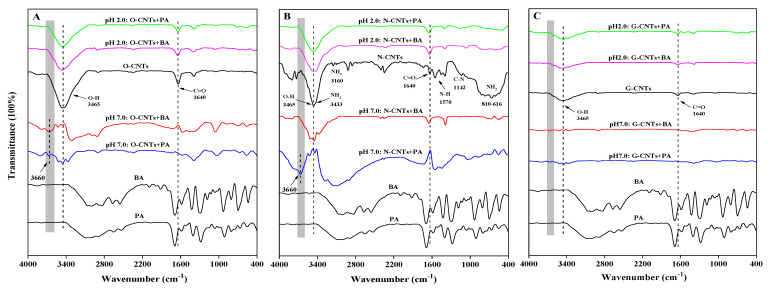
FTIR spectra analysis of before and after adsorption of BA and PA on the O-CNTs (**A**), N-CNTs (**B**) and G-CNTs (**C**) at pH 2.0 and 7.0, respectively. A clearly new peak appeared at the higher frequency (3660 cm^−1^, the blue shift of O−H or –NH_2_) reveals the formation of the homonuclear CAHB or heteronuclear CAHB.

Previous studies indicated that the blue shift of the characteristic peaks in FTIR (moving to higher frequencies) revealed the special strong H-bond with rising intensity and curtate distance between the donor and acceptor [50,51], which was confirmed in our previous work [18]. As shown in Figure 5A, two obvious characteristic peaks for O-CNTs before adsorption were observed attributed to 3465 cm^−1^ (O-H) and 1640 cm^−1^ (C=O), respectively. After BA or PA adsorption, although the intensity of the above two characteristic peaks on O-CNTs slightly decreased at pH 2.0, the position of the absorption peaks did not change. This result indicates that BA and PA could interact with the O-H and C=O functional groups on O-CNTs surface by ordinary H-bond. However, at pH 7.0, these two peaks (3465 cm^−1^ and 1640 cm^−1^) have undergone obvious changes or even lost after O-CNTs adsorbing BA or PA. Meanwhile, a new peak with a higher frequency (3660 cm^−1^) emerged, which could be attributed to the strong homonuclear CAHB formation between BA/PA and O-CNTs, resulting in the blue shift of the −OH peak.

For N-CNTs (Figure 5B), after adsorption of BA or PA, the characteristic peaks NH_2_ (3433, 3160, 810~616 cm^−1^) and N-H (1570 cm^−1^) of N-CNTs all disappeared at pH 2.0, indicating that BA and PA also could interact with these functional groups of N-CNTs by ordinary H-bonds. What is noteworthy is that, at pH 7.0, there was no new peak arose in the high frequency band after adsorption of BA, while a new peak (3660 cm^−1^) did appear in the high-frequency band after adsorption of PA. This may be because the larger ∆p*K*_a_ ≈ 4.59 (the difference is almost close to 5) between BA and N-CNTs leads to the formation of heteronuclear CAHB more likely to be an ordinary H-bond. Compared with BA, the smaller ∆p*K*_a_ ≈ 3.48 between PA and N-CNTs makes the formation of heteronuclear CAHB much easier and stronger. For G-CNTs, due to the low oxygen content (0.22%, Table S1), there was no significant change in the characteristic peaks at pH 2.0 after adsorption of BA or PA, and there was also no blue shift H-bond that appeared at pH 7.0. These results are consistent with our above discussion in Section 3.3 and Section 3.4, which further confirms that CAHB is an important mechanism for the adsorption of BA/PA on O-CNTs and N-CNTs. However, it is difficult to quantitatively analyze the bond strength and adsorption energy of homonuclear/heteronuclear CAHB on the molecular scale by using traditional spectral methods. DFT calculation can provide a deeper insight into the H-bond between CNTs and IOCs [29,52]. Therefore, DFT calculation is further employed to analyze the strength of homonuclear/heteronuclear CAHB formed between CNTs and BA/PA.

**Figure 6:**
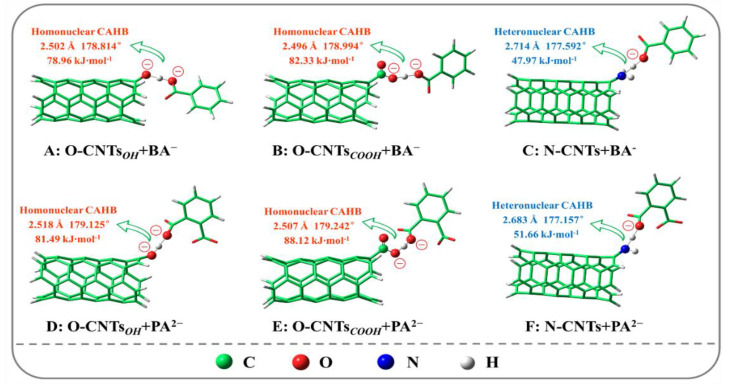
The Binding energy (E_ads_, kJ·mol^−1^), bond length (Å) and bond angle (°) of charged assisted H-bonds (CAHBs) between CNTs and IOCs. The shorter bond length and the closer linear bond angle (180°) indicate the stronger H-bond, and the strength order of CAHBs is Homonuclear CAHB (**A**,**B**,**D**,**E**) > Heteronuclear CAHB (**C**,**F**).

Figure 6 shows the optimized structures and calculation models of homonuclear/hetero nuclear CAHB formed by O-CNTs and N-CNTs adsorbing BA or PA at pH 7.0. Table 1 displays the binding energy, bond length, and bond angle of homonuclear/heteronuclear CAHB. As we all know, the H-bond is more powerful when the bond length is shorter, and the bond angle is nearer to linearity (180°). For O-CNTs, homonuclear CAHB was formed with BA (CNTs-Oˉ···H+···ˉOOC-BA, −78.96 kJ·mol^−1^, 178.814°, 2.502 Å; CNTs-COOˉ···H+···ˉOOC-BA, −82.33 kJ·mol^−1^, 178.994°, 2.496 Å) and PA (CNTs-Oˉ···H+···ˉOOC-PA, −81.49 kJ·mol^−1^, 179.125°, 2.518 Å; CNTs-COOˉ···H+···ˉOOC-PA, −88.12 kJ·mol^−1^, 179.242°, 2.507 Å). For N-CNTs, heteronuclear CAHB was formed with BA (CNTs-NH2···H+···ˉOOC-BA, −47.97 kJ·mol^−1^, 177.592°, 2.714 Å) and PA (CNTs-NH2···H+···ˉOOC-PA, −51.66 kJ·mol^−1^, 177.157°, 2.683 Å). Obviously, compared with heteronuclear CAHB, homonuclear CAHB has higher adsorption energy, a shorter bond length, and a closer linear bond angle. It is worth noting that the heteronuclear CAHB between PA and N-CNTs is stronger than that between BA and N-CNTs. This is due to the smaller ∆pK_a_≈3.48 between N-CNTs and PA relative to BA (∆pK_a_≈4.59), which is consistent with the above description and conclusion. In conclusion, all the spectral evidence and DFT calculations indicate that homonuclear/heteronuclear CAHB plays an essential role in the adsorption of BA/PA by functionalized CNTs. Importantly, homonuclear CAHB is stronger than heteronuclear CAHB.

## 4. Conclusions and Environmental Significance

In this study, the adsorption and desorption behaviors of BA and PA on three CNTs at different pHs were explored. The adsorption mechanism was discussed in detail by combining it with DFT simulation and FTIR characterization. The kinetics data implied that the adsorption of BA and PA on O-CNTs was mainly a chemical process at pH 7.0, especially and the negatively charged BA and PA could form strongly homonuclear CAHB with O-groups on the O-CNTs surface. For the adsorption isotherms, the Freundlich model was more appropriate than the Langmuir model, indicating BA and PA were more preferred to adsorb on the CNTs’ surface. Under different pH conditions, the adsorption order of BA and PA on three CNTs was various. Especially at pH 4.0 and 7.0, the adsorption capacity of BA/PA on O-CNTs was clearly higher than that on N-CNTs, which can be attributed to CAHB formation, and the strength of homonuclear CAHB (≥78.96 kJ·mol^−1^) was stronger than that of heteronuclear CAHB (≤51.66 kJ·mol^−1^). In desorption assays under pH 7.0, the strong homonuclear CAHB formed between BA/PA and O-CNTs, leading to a clear desorption hysteresis phenomenon (HI ≥ 3.51). Compared with the homonuclear CAHB, the relatively weaker heteronuclear CAHB between BA/PA and N-CNTs resulted in less significant desorption hysteresis (HI ≥ 1.47). Importantly, it was found that the contribution of homonuclear CAHB (BA: 54.17% and PA: 52.70%) was greater than that of heteronuclear CAHB (BA: 42.55% and PA: 45.79%) at pH 7.0. The above conclusions were further confirmed by FTIR characterization and DFT calculation, and the key evidence of CAHB in FTIR was found. Moreover, DFT calculation also provided pivotal information on the bond length and bond angle of homonuclear/heteronuclear CAHB at the molecular scale, which further revealed that homonuclear CAHB is stronger. Considering that different species’ proportions of IOCs at different pHs would lead to different adsorption mechanisms, especially the role of CAHB should be carefully evaluated. In this study, DFT calculation and experimental tests were used to compare the strength of homonuclear/heteronuclear CAHB, which provides a new light for better comprehending the interaction between IOCs and carbonaceous materials. Moreover, the combination of DFT calculation, FTIR, and experiments would provide a new platform for studying the adsorption behavior between carbonaceous adsorbents and other organic and inorganic pollutants.

## Figures and Tables

**Table 1:** The binding energy (Eads, kJ·mol^−1^), bond length (Å) and bond angle (°) of charged assisted H-bonds for BA and PA adsorption on O-CNTs and N-CNTs.

Adsorption Configurations	Eads (kJ·mol^−1^)	Bond Length (Å)	Bond Angle (°)
O-CNTsOH···BA^−^	−78.96	2.502	178.814
O-CNTsCOOH···BA^−^	−82.33	2.496	178.994
N-CNTs···BA^−^	−47.97	2.714	177.592
O-CNTsOH···PA^2−^	−81.49	2.518	179.125
O-CNTsCOOH···PA^2−^	−88.12	2.507	179.242
N-CNTs···PA^2−^	−51.66	2.683	177.157

## Data Availability

Further information can be obtained from the corresponding author.

## Supplementary Materials

The following supporting information can be downloaded at: https://www.mdpi.com/article/10.3390/ijerph191912118/s1: Table S1. Physicochemical properties of four different functionalized CNTs; Table S2. Selected physicochemical properties of BA and PA; Table S3. Kinetic fitting parameters of the pseudo-first order, pseudo-second order and intra-particle diffusion models for adsorption of BA and PA on O-CNTs; Table S4. Fitting parameters of Freundlich, Langmuir and Dubinin–Radushkevich models for BA and PA on CNTs at different pHs; Table S5. Release ratio (R_r_, %) and hysteresis index of BA and PA from CNTs after three desorption steps at 298 K and various concentration (Low, Middle and High) of adsorbates. Low, Middle and High represent 5 mg·L^−1^, 80 mg·L^−1^ and 160 mg·L^−1^ of BA/PA; Figure S1. Adsorption equilibrium time of BA and PA onto O-CNTs at different pHs. The initial concentration was 50 mg·L^−1^ for adsorbates. There is no significant difference in Q_e_ after 48 h. Thus, we consider that 48 h is long enough to achieve adsorption equilibrium; Figure S2. Optimized structures of CNTs (A: O-CNTs-_OH_, B: O-CNTs-_COOH_ C: N-CNTs), BA (D) and PA (E). Figure S3. TEM and SEM images of O-CNTs (A and D), N-CNTs (B and E) and G-CNTs (C and F), respectively; Figure S4. Pore size distributions of O-CNTs, N-CNTs and G-CNTs, respectively. Figure S5. Peak fitting of O1s for O-CNTs; Figure S6. FTIR spectra of O-CNTs, N-CNTs and G-CNTs at room temperature at the range of 400~4000 cm^−1^; Figure S7. The speciation distribution diagram of BA and PA in water at different pHs; Figure S8. Adsorption and desorption of PA on O-CNTs, N-CNTs and G-CNTs at pH 2.0 (A, B and C) and pH 7.0 (D, E and F), respectively. And the comparison of the hysteresis index of PA (low, middle and high represent 5, 80 and 160 mg·L^−1^ of PA, respectively) adsorption on three types of CNTs (G, H and I) at pH 2.0 and pH 7.0. At pH2.0, there is no obvious desorption hysteresis of PA on three CNTs, but the obvious desorption hysteresis of PA on O-CNTs and N-CNTs occurred at pH 7.0. The hysteresis index of PA on O-CNTs (≥3.51) and N-CNTs (≥2.60) is higher than that of BA on G-CNTs (≤0.94) at pH 7.0. References [53,54] are mentioned in Supplementary Materials.
