# Supplementary material for: Effect of pH-Dependent Homo/Heteronuclear CAHB on Adsorption and Desorption Behaviors of Ionizable Organic Compounds on Carbonaceous Materials
Source: Int J Environ Res Public Health. 2022 Sep 25;19(19):12118. doi: 10.3390/ijerph191912118 (PMC9566536; doi:10.3390/ijerph191912118)
Supplement: Supplementary file 1 [file ijerph-19-12118-s001.zip › ijerph-1906580-SM.pdf]

Supporting information

Effect of pH-dependent homo/heteronuclear CAHB on adsorption and desorption behaviors of ionizable organic compounds on carbonaceous materials

Xiaoyun Li [a,b,*], Jinlong Zhang [a], Yaofeng Jin [a], Yifan Liu [a], Nana Li [a], Yue Wang [a], Cong Du [a], Zhijing Xue [a], Nan Zhang [c], Qin Chen [d,*]

[a] *Department of Environmental Science, School of Geography and Tourism, Shaanxi Normal University, Xi'an 710119, China*

[b] *International Joint Research Centre of Shaanxi Province for Pollutants Exposure and Eco-environmental Health, Xi'an 710119, China*

[c] *Environmental Protection Department of Mahe Town, Yuyang District, Yulin 719000, China*

[d] *Northwest Land and Resource Research Center, Shaanxi Normal University, Xi'an 710119, China*

[*]Corresponding authors

E-mail address: lxy518@snnu.edu.cn (Dr. Xiaoyun Li); chenqin@snnu.edu.cn (Dr. Qin Chen)

Number of Pages: 17

Number of Texts: 2

Number of Tables: 5

Number of Figures: 8

Text S1: The TEM and SEM, Total elemental composition and XPS, BET, FTIR, Buffering capability and pH_{PZC} characterization procedures of CNTs.

TEM and SEM: For TEM, after dispersing CNTs with ethanol, ultrasonic was used for about 60 min. Using droppers to absorb the suspension, drop by drop on the carbon film supported by copper mesh. Then the morphology was observed by TEM (JEM-2100, Japan) after drying with fluorescent lamp. For SEM, the CNTs were directly placed on the conductive adhesive, and the morphology characteristics were observed by scanning electron microscope (SEM, FEI Quanta 200, USA) after fixation.

Total elemental composition and XPS: The total elemental composition of CNTs was determined by elemental analyzer (VarioELIII, Germany). CNTs were put into the oven with drying at 40℃ for 6 h, and then CNTs (about 5 mg) were weighted on a known weight of tinfoil paper. Wrap it in tin foil and weigh/record the qualities after squeezing out the air with tweeze. CHNS mode was selected for instrument measurement. The surface elemental composition was determined by X-ray photoelectron spectroscopy (XPS, AXIS-ULTRA, Japan) and the scanning range was 0~1200 eV. The instrument used the C1s peak of carbon to correct the electron binding energy. First, the full spectrum scan was carried out on the CNTs, and then the required elements such as carbon, oxygen and nitrogen were scanned respectively. XPSPEAK software was used for peak processing and detailed spectrogram analysis of each element.

Surface area (BET) and pore size distributions: In this study, the specific surface area and pore size distribution analyzer (ASAP2460, Micromeritics, USA) was used to measure the N_2 adsorption-desorption curve at 77 K, and the specific surface area and pore size distribution of the CNTs were tested and analyzed. CNTs should be degassed at 200 ℃ for 2 h before determination. The specific surface area was calculated by Brunauer-Emmett-Teller (BET) method, and the pore size distribution was calculated by Barrett-Joyner-Halenda (BJH) method.

FTIR: The FTIR spectra of the samples were tested on Tensor 27 (Bruker, Germany) infrared spectrometer using KBr tableting method at room temperature. The determination range was 4000~400 cm^{-1}. Each sample was scanned 100 times, and the spectrograms were processed by OMNIC and Origin softwares.

Buffering capability and pH_{PZC}: For Buffering capability, the weigh 0.05 g CNTs into 20 mL brown glass bottle with Teflon screw cap, add 20 mL background solution with known pH (2.0-12.0). After shaking for 48h in a constant temperature shock chamber (25°C), the sample was centrifuged to determine the pH values of the supernatant. Meanwhile, the known pH background solution without CNTs was used as the control, and the same treatment was carried out. The Buffering capability of CNTs was determined according to the change of pH values of background solution before and after experiment. For pH_{PZC}, different from the above buffering capability steps, the ξ -potential (mV) was measured directly with a Nano Zeta Sizer (Bi-90 plus, Brookhaven, USA) after shaking for 48h (centrifugation was not required) at different pH conditions (2.0-10.0). The determinations were conducted at least three times.

Text S2: The analysis procedures of adsorption kinetics, adsorption isotherms and distribution coefficient (K_d).

Pseudo-first-order, pseudo-second-order and Weber–Morris intra-particle diffusion model were used to fit the adsorption kinetic data:

Pseudo-first-order kinetic model:

$$\text{In}(Q_e - Q_t) = \ln Q_e - k_1 t \quad \text{(S1)}$$

Pseudo-second-order kinetic model:

$$\frac{t}{Q_t} = \frac{1}{k_2 Q_e} + \frac{t}{Q_e} \quad \text{(S2)}$$

Intra-particle diffusion model:

$$Q_t = k_i t^{0.5} + C \quad \text{(S3)}$$

Where Q_e and Q_t (mg·g^{-1}) are the equilibrium adsorbed concentration and the adsorbed concentration at time t (h, the contact time), k_1 (h^{-1}) and k_2 (g·mg^{-1}·h^{-1}) are the adsorption rate constant, k_i (mg·g^{-1}·h$^{-0.5}$) and C are the intra-particle diffusion rate constant and intercept, respectively.

The Freundlich and Langmuir models were used to fit the adsorption data:

Freundlich model:

$$Q_e = K_f C_e^{1/n} \quad \text{(S4)}$$

Langmuir model:

$$Q_e = Q_0 C_e / (K_L + C_e) \quad \text{(S5)}$$

Where Q_e (µmol·g^{-1}·m^{-2}) is the equilibrium adsorbed concentration, C_e (mmol·L^{-1}) is the equilibrium solution-phase concentration, K_f ((µmol·g^{-1})/(mmol·L^{-1})^n) is the Freundlich affinity-capacity coefficient, and n is the Freundlich exponential coefficient. Q_0 (µmol·g^{-1}·m^{-2}) is the maximum adsorption capacity of monolayer, K_L (mmol·L^{-1}) is the Langmuir equilibrium constant, which is related to the properties and temperature of adsorbent and adsorbate.

The adsorption affinity of IOCs on CNTs could be described through the distribution coefficient (K_d):

$$K_d = Q_e / C_e \quad \text{(S6)}$$

Where Q_e (mg·kg^{-1}) and C_e (mg·L^{-1}) are the equilibrium adsorption concentration and equilibrium solution-phase concentration.

Table S1. Physicochemical properties of four different functionalized CNTs

Sample Properties	Purity (%)	Ash (wt%)	XPS (atom based%) C	O	N	ID[d] (nm)	OD[e] (nm)	A_{surf}[f] ($m^2·g^{-1}$)	Pore size[g] (nm)	Pore volume[h] (cm^3/g)	PZC[i]
O-CNTs[a]	>99.9	<0.1	94.29	5.71	0	3-5	8-15	81.50	27.46	0.56	3.65
N-CNTs[b]	>95.0	<5.0	96.46	2.56	0.98	3-5	8-15	172.61	26.43	1.02	8.76
G-CNTs[c]	>99.9	<0.1	99.78	0.22	0	3-5	8-15	66.87	30.13	0.48	--

[a]: Oxygen-enriched carbon nanotubes; [b]: Amino-enriched carbon nanotubes; [c]: Graphitized carbon nanotubes; [d]: Inner diameter of the CNTs; [e]: Outer diameter of the CNTs; [f]: Surface area was measured by multipoint B.E.T method; [g]: pore size was total adsorption average pore width (4V/A by BET, A is the surface area obtained by B.E.T method); [h]: pore volume was BJH adsorption cumulative volume with the pore diameter range of 2-150 nm; [i]: The point of zero charge of adsorbents were measured by a Nano Zeta sizer. The length of the three CNTs used in this study is 50 nm.

Table S2. Selected physicochemical properties of of BA and PA

Compounds	Structures[a]	MW[b] (g/mol)	pK_a[c]	LogK_{ow}[d]	S_w[e] g/L
Benzoic acid (BA)	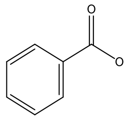	122.12	pK_a = 4.17[1,2]	pH 2.0: 1.63[3]<br>pH 4.0: 1.37[3]<br>pH 7.0: -1.20[3]	pH 2.0: 4.34[3]<br>pH 7.0: 430.55[3]
Phthalic acid (PA)	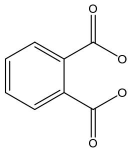	166.13	pK_{a1} = 2.98[1,2]<br>pK_{a2} = 5.28[1,2]	pH 2.0: 1.24[3]<br>pH 4.0: 0.18[3]<br>pH 7.0: -3.65[3]	pH 2.0: 4.55[3]<br>pH 7.0: 408.54[3]

[a]: structures (https://pubchem.ncbi.nlm.nih.gov/; accessed on May 4th, 2020); [b]: Molecular weight; [c]: Acid dissociation constants; [d]: n-Octanol/water partition coefficients; [e]: Water solubility of sorbates.

[1] Nagayasu, T.; Yoshioka, C.; Imamura, K.; Nakanishi, K. Effects of carboxyl groups on the adsorption behavior of low-molecular-weight substances on a stainless steel surface. *Journal of Colloid and Interface Science* **2004**, 279 (2): 296-306.

[2] Li, X. Y.; Pignatello, J. P.; Wang, Y. Q.; Xing, B. S. New Insight into Adsorption Mechanism of Ionizable Compounds on Carbon Nanotubes. *Environmental Science & Technology* **2013**, 47(15): 8334-8341.

[3] https://chemicalize.com/

Table S3. Kinetic fitting parameters of the pseudo-first order, pseudo-second order and intra-particle diffusion models for adsorption of BA and PA on O-CNTs

Adsorbents	$Q_{e,exp}$ (mg·g^{-1})	Pseudo-first order				Pseudo-second order				Intra-particle diffusion			
		k_1[a]	$Q_{e,cal}$ (mg·g^{-1})	Adj r^2[b]	SSR[c]	k_2[d]	$Q_{e,cal}$ (mg·g^{-1})	Adj r^2	SSR	k_{id}[e]	C (mg·g^{-1})	Adj r^2	SSR
BA	2.29	0.01	1.32	0.6066	18.432	8.71	2.34	0.9998	0.758	0.13	1.38	0.7376	1.636
PA	2.96	0.03	0.36	0.2192	27.921	70.95	3.00	0.9997	0.953	0.11	2.26	0.4622	3.492

[a] h^{-1}; [b] Adjusted square of correlation coefficient; [c] Sum of squares residual; [d] g·mg^{-1}·h^{-1}; [e] mg·g^{-1}·h$^{-1/2}$

Table S4. Fitting parameters of Freundlich, Langmuir and Dubinin-Radushkevich models for BA and PA on CNTs at different pHs.

Sorbates	pH	Sorbents	Freundlich model				Langmuir model			
			K_f	1/n	Adj r^2	SSR	Q_0 ($\mu mol \cdot g^{-1} \cdot m^{-2}$)	K_L	Adj r^2	SSR
BA	pH 2.0	O-CNTs	1.32±0.04	0.39±0.03	0.9811	0.0358	1.47±0.07	0.15±0.02	0.9871	0.0581
		N-CNTs	1.22±0.02	0.44±0.02	0.9951	0.0054	1.43±0.01	0.22±0.05	0.9745	0.0281
		G-CNTs	1.45±0.05	0.39±0.03	0.9762	0.0018	1.71±0.07	0.19±0.02	0.9889	0.0223
	pH 4.0	O-CNTs	2.12±0.07	0.51±0.03	0.9826	0.0025	2.66±0.14	0.32±0.04	0.9900	0.0361
		N-CNTs	0.20±0.01	0.39±0.02	0.9858	0.0005	0.23±0.02	0.18±0.06	0.9206	0.0026
		G-CNTs	1.86±0.03	0.53±0.02	0.9931	0.0213	2.65±0.15	0.46±0.05	0.9891	0.0334
	pH 7.0	O-CNTs	0.51±0.01	0.71±0.04	0.9915	0.0015	1.27±0.24	1.49±0.42	0.9853	0.0026
		N-CNTs	0.23±0.01	0.87±0.05	0.9905	0.0005	2.00±1.54	6.64±5.81	0.9862	0.0007
		G-CNTs	0.22±0.02	0.58±0.04	0.9669	0.0006	0.27±0.03	0.37±0.09	0.9623	0.0008
PA	pH 2.0	O-CNTs	1.51±0.13	0.48 ±0.05	0.9578	0.0081	1.14±0.03	0.08±0.01	0.9953	0.0342
		N-CNTs	1.56±0.13	0.54 ±0.05	0.9488	0.0049	1.29±0.06	0.10±0.01	0.9901	0.0106
		G-CNTs	1.74±0.09	0.43±0.03	0.9779	0.0031	1.30±0.06	0.06±0.01	0.9841	0.0166
	pH 4.0	O-CNTs	0.99±0.05	0.41±0.03	0.9747	0.0112	0.81±0.03	0.08±0.01	0.9859	0.0162
		N-CNTs	0.90±0.04	0.45±0.02	0.9877	0.0047	0.80±0.07	0.13±0.03	0.9736	0.0100
		G-CNTs	1.16±0.06	0.43±0.03	0.9709	0.0177	0.98±0.06	0.10±0.02	0.9669	0.0201
	pH 7.0	O-CNTs	0.60±0.05	0.65±0.07	0.9501	0.0045	0.73±0.09	0.45±0.10	0.9821	0.0103
		N-CNTs	0.67±0.03	1.82±0.09	0.9956	0.0006	3.92E07±1.92E14	9.52E07±4.66E14	0.8696	0.0013
		G-CNTs	0.23±0.02	1.13±0.02	0.9756	0.0003	0.89E07±2.03E13	4.28E07±9.77E14	0.9802	0.0014

Table S5. Release ratio (R_r, %) and hysteresis index of BA and PA from CNTs after three desorption steps at 298 K and various concentration (Low, Middle and High) of adsorbates. Low, Middle and High represent 5 mg/L, 80 mg/L and 160 mg/L of BA/PA.

pH	Adsorbents	BA (Release ratio, %)			PA (Release ratio, %)			BA (Hysteresis index)			PA (Hysteresis index)		
		Low	Middle	High	Low	Middle	High	Low	Middle	High	Low	Middle	High
2.0	O-CNTs	39.20	39.68	38.79	36.85	35.53	36.31	0.16	0.17	0.23	0.09	0.23	0.22
	N-CNTs	39.99	35.60	40.26	42.54	46.17	47.19	0.03	0.01	0.02	0.02	0.02	0.21
	G-CNTs	46.02	40.60	52.50	56.69	40.70	44.74	0.07	0.08	0.02	0.02	0.01	0.02
7.0	O-CNTs	2.48	3.66	3.58	3.87	1.75	2.25	5.59	5.29	4.12	3.92	4.34	3.51
	N-CNTs	11.78	15.30	17.51	10.52	8.16	9.90	1.47	1.60	2.17	3.10	2.60	2.94
	G-CNTs	59.27	58.68	54.28	56.48	52.29	57.20	0.94	0.88	0.99	0.94	0.85	0.65

Text: CAHB contribution at pH 7.0

(1) BA (Calculated by the R_r average values of low, middle and high concentration)

Homonuclear CAHB: $R_r^{G\text{-}CNTs} - R_r^{O\text{-}CNTs} = 54.17\%$

Heteronuclear CAHB: $R_r^{G\text{-}CNTs} - R_r^{N\text{-}CNTs} = 42.55\%$

(2) PA (Calculated by the R_r average values of low, middle and high concentration)

Homonuclear CAHB: $R_r^{G\text{-}CNTs} - R_r^{O\text{-}CNTs} = 52.70\%$

Heteronuclear CAHB: $R_r^{G\text{-}CNTs} - R_r^{N\text{-}CNTs} = 45.79\%$

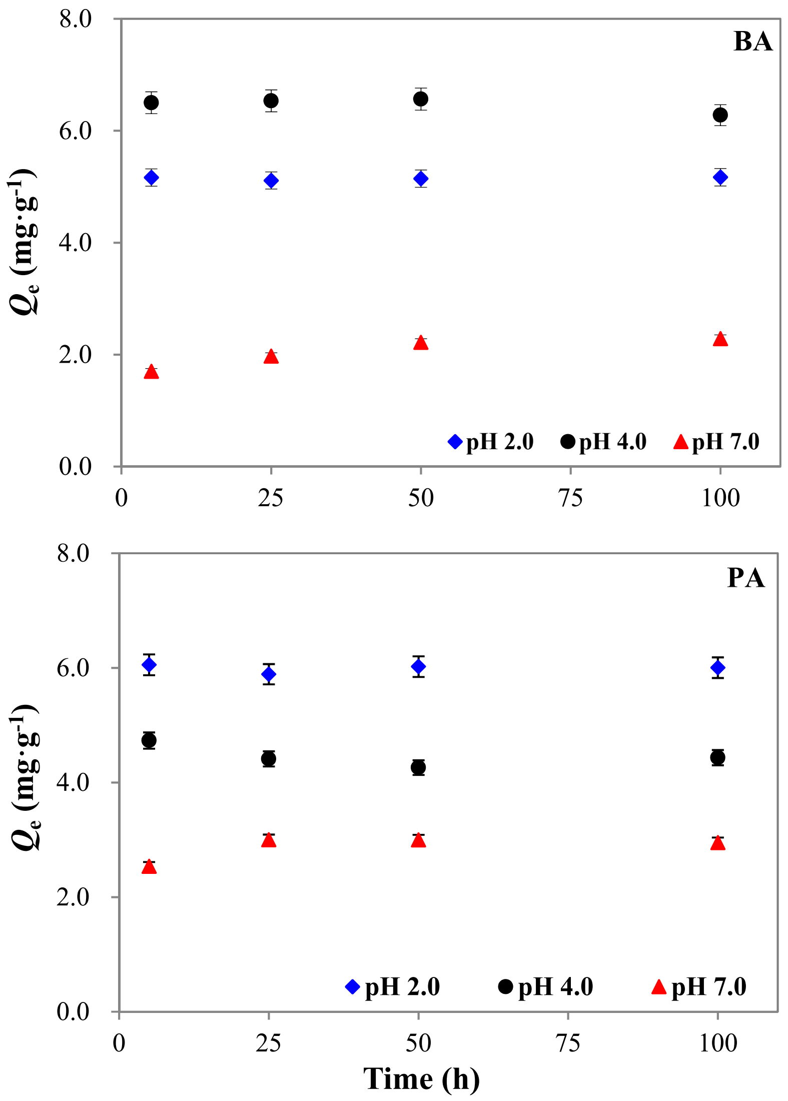

Fig. S1. Adsorption equilibrium time of BA and PA onto O-CNTs at different pHs. The initial concentration was 50 mg·L^{-1} for adsorbates. There is no significant difference in Q_e after 48 h. Thus, we consider that 48 h is long enough to achieve adsorption equilibrium.

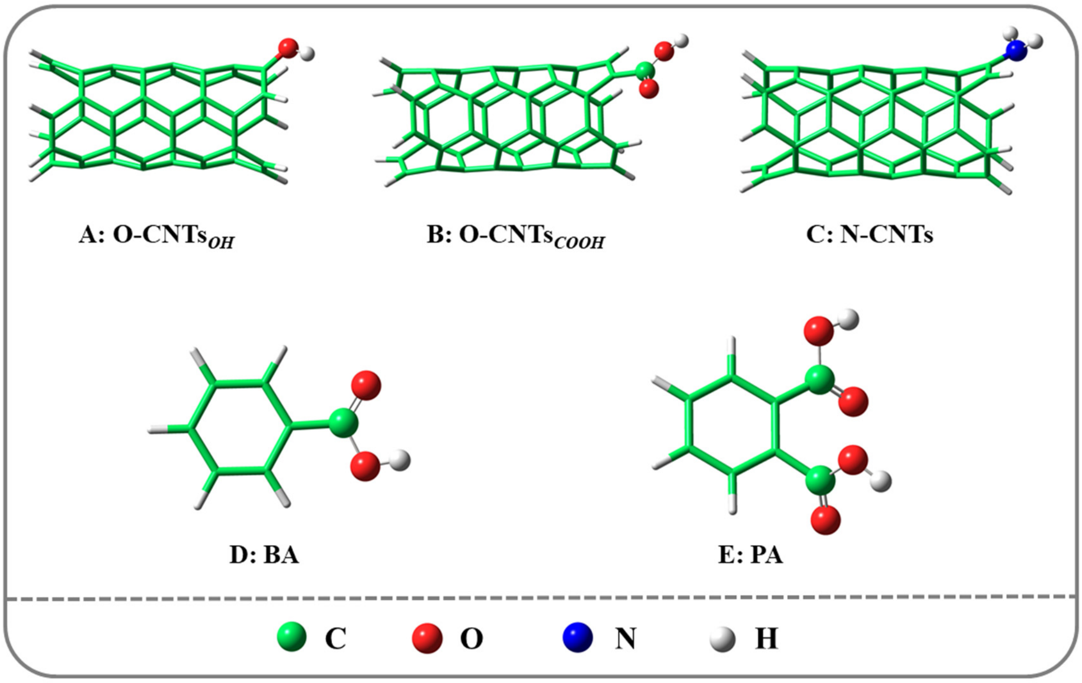

Fig. S2. Optimized structures of CNTs (A: O-CNTs$_{OH}$, B: O-CNTs$_{COOH}$ C: N-CNTs), BA (D) and PA (E).

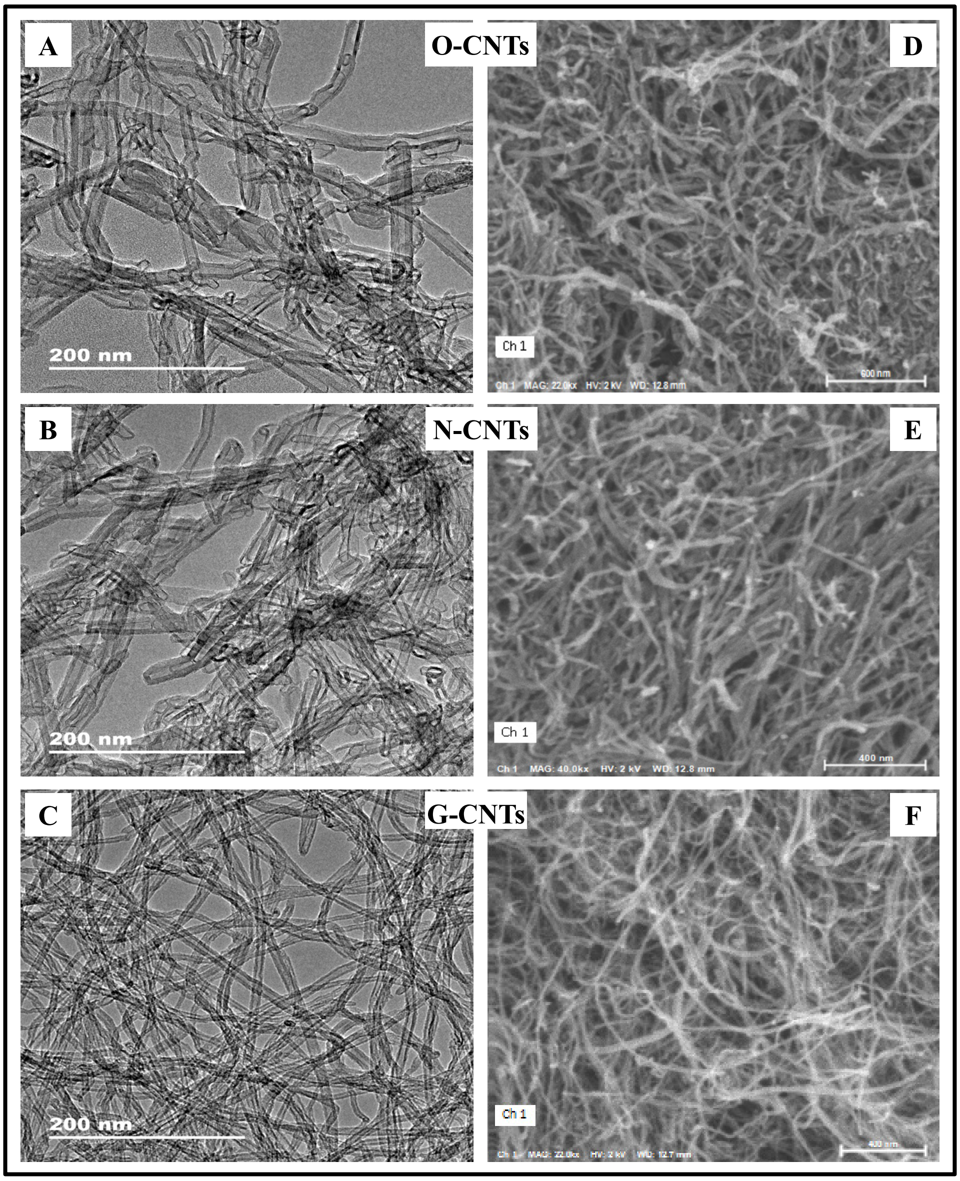

Fig. S3. TEM and SEM images of O-CNTs (A and D), N-CNTs (B and E) and G-CNTs (C and F), respectively.

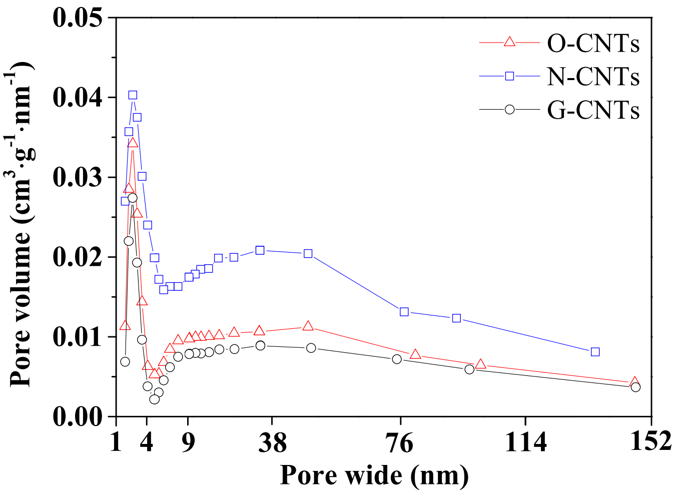

Fig. S4. Pore size distributions of O-CNTs, N-CNTs and G-CNTs, respectively.

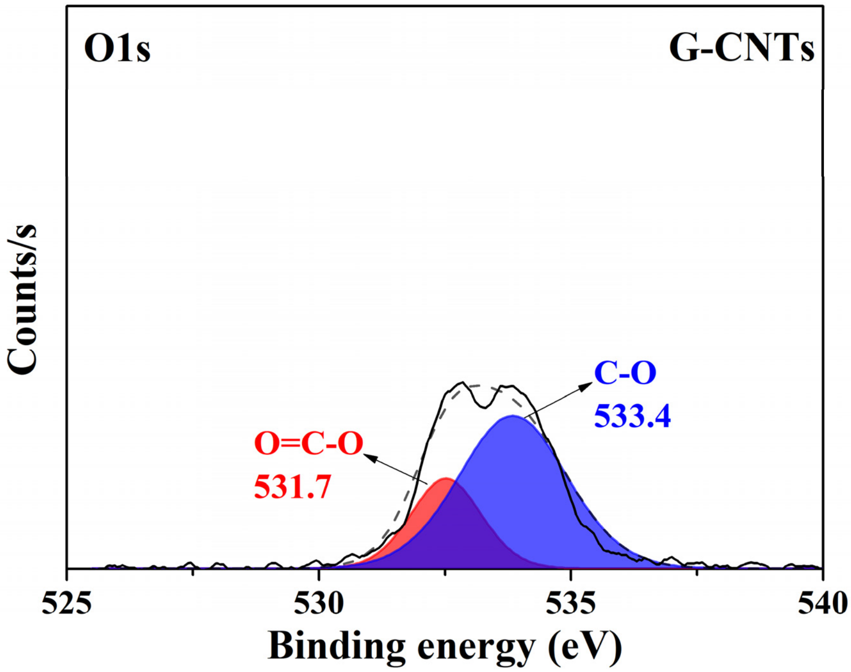

Fig. S5. Peak fitting of O1s for O-CNTs.

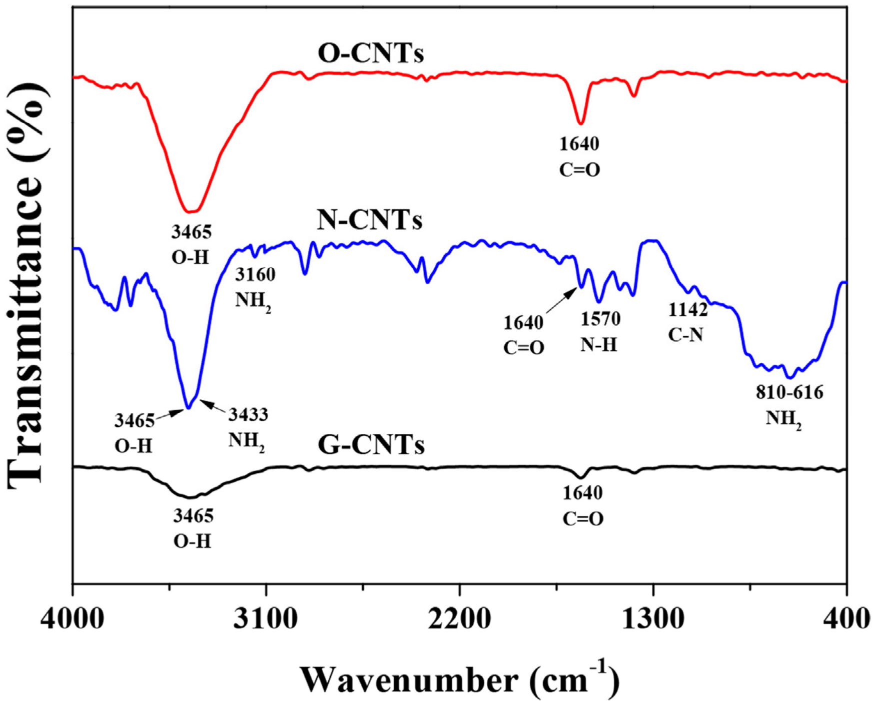

Fig. S6. FTIR spectra of O-CNTs, N-CNTs and G-CNTs at room temperature at the range of 400~4000 cm^{-1}.

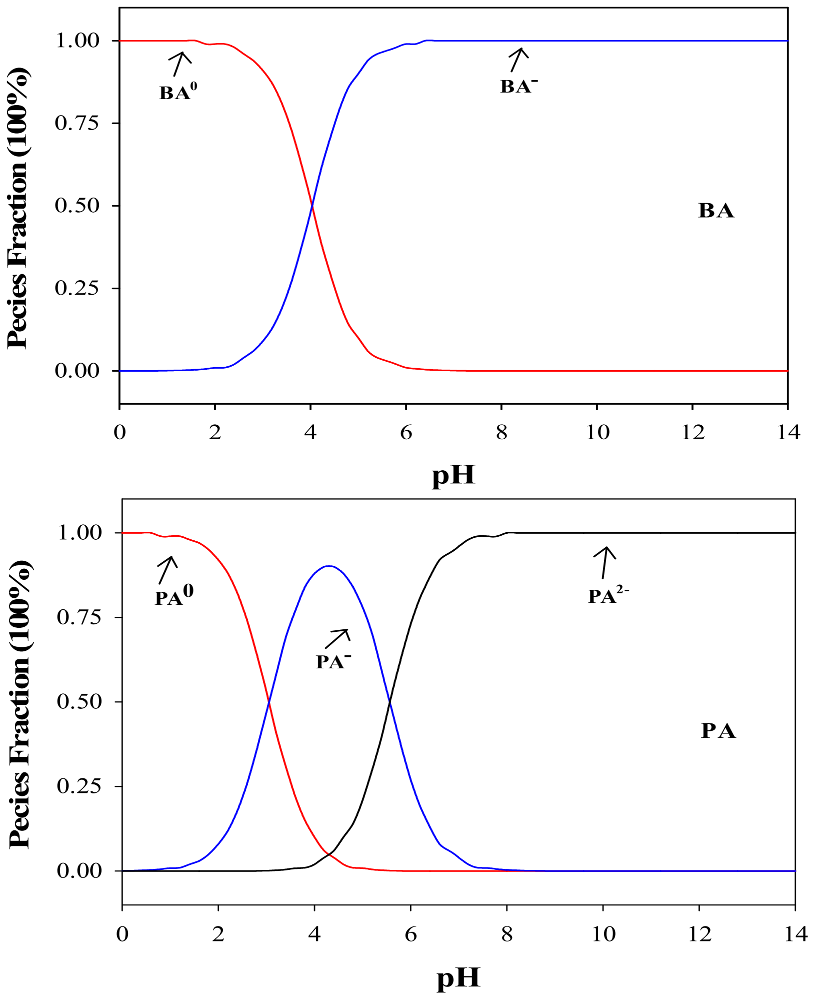

Fig. S7. The speciation distribution diagram of BA and PA in water at different pHs.

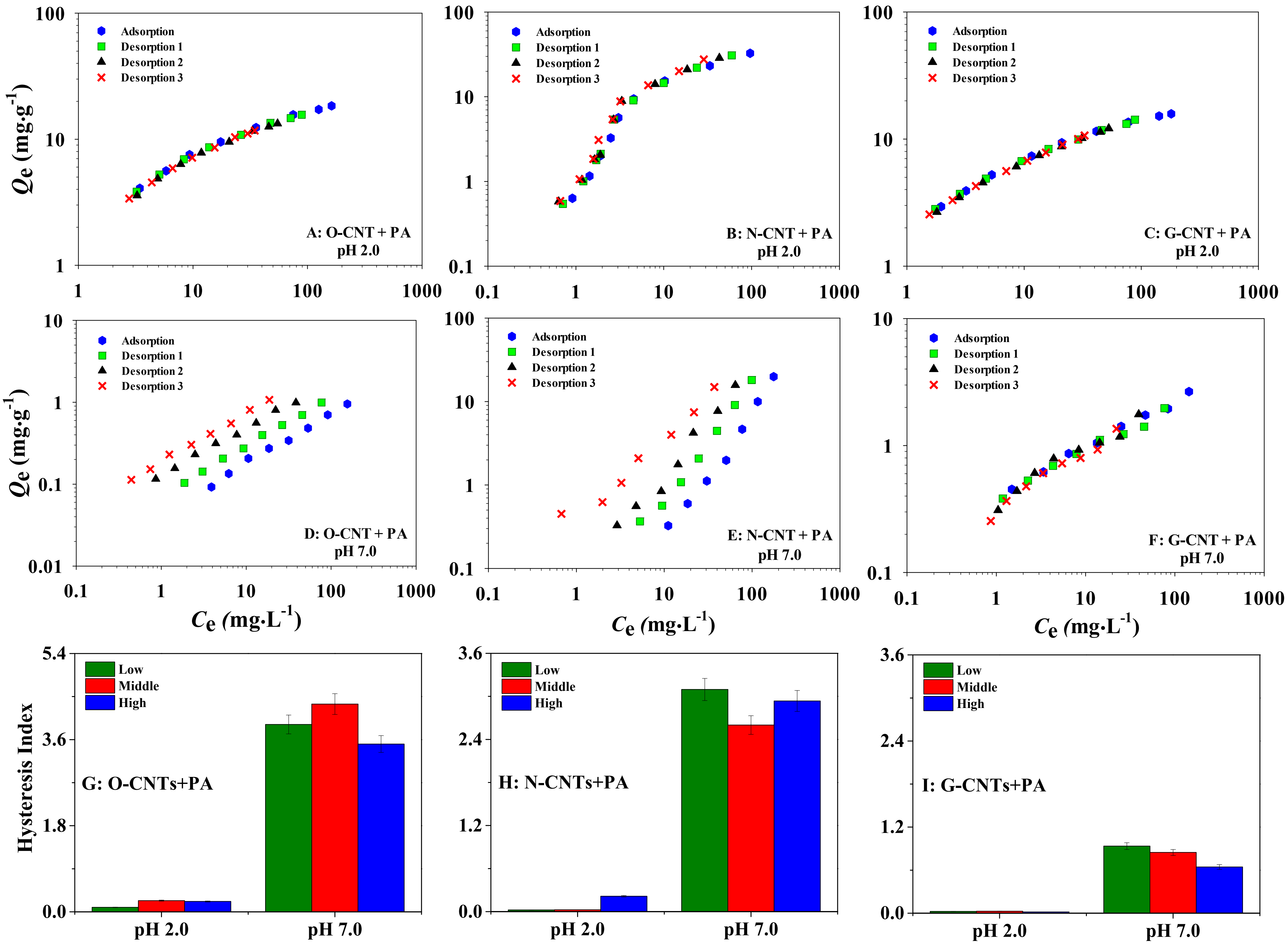

Fig. S8. Adsorption and desorption of PA on O-CNTs, N-CNTs and G-CNTs at pH 2.0 (A, B and C) and pH 7.0 (D, E and F), respectively. And the comparison of the hysteresis index of PA (Low, Middle and High represent 5 mg/L, 80 mg/L and 160 mg/L of PA) adsorption on three types of CNTs (G, H and I) at pH 2.0 and pH 7.0. At pH2.0, there is no obvious desorption hysteresis of PA on three CNTs, but the obvious desorption hysteresis of PA on O-CNTs and N-CNTs occurred at pH 7.0. The hysteresis index of PA on O-CNTs (≥ 3.51) and N-CNTs (≥ 2.60) is higher than that of BA on G-CNTs (≤ 0.94) at pH 7.0.
